# Circulating cell-free microRNA as biomarkers for screening, diagnosis and monitoring of neurodegenerative diseases and other neurologic pathologies

**DOI:** 10.3389/fncel.2013.00150

**Published:** 2013-09-10

**Authors:** Kira S. Sheinerman, Samuil R. Umansky

**Affiliations:** DiamiR, LLCPrinceton, NJ, USA

**Keywords:** miRNA, neurodegeneration, Alzheimer's disease, neurologic pathology, screening, biomarker, plasma, CSF

## Abstract

Many neurodegenerative diseases, such as Alzheimer's disease, Parkinson disease, vascular and frontotemporal dementias, as well as other chronic neurological pathologies, are characterized by slow development with a long asymptomatic period followed by a stage with mild clinical symptoms. As a consequence, these serious pathologies are diagnosed late in the course of a disease, when massive death of neurons has already occurred and effective therapeutic intervention is problematic. Thus, the development of screening tests capable of detecting neurodegenerative diseases during early, preferably asymptomatic, stages is a high unmet need. Since such tests are to be used for screening of large populations, they should be non-invasive and relatively inexpensive. Further, while subjects identified by screening tests can be further tested with more invasive and expensive methods, e.g., analysis of cerebrospinal fluid or imaging techniques, to be of practical utility screening tests should have high sensitivity and specificity. In this review, we discuss advantages and disadvantages of various approaches to developing screening tests based on analysis of circulating cell-free microRNA (miRNA). Applications of circulating miRNA-based tests for diagnosis of acute and chronic brain pathologies, for research of normal brain aging, and for disease and treatment monitoring are also discussed.

## INTRODUCTION

There are several basic types of clinical tests: (i) genetic tests that help predict predisposition to a particular disease; (ii) screening tests, which are applied to a large population for early detection of a disease, preferably prior to its clinical manifestation; (iii) diagnostic tests, which are applied when a person has clinical symptoms of a disease or when the pathology has been detected by a screening test; (iv) predictive tests aimed to predict the disease outcome and drug sensitivity; and (v) disease and treatment monitoring tests. Screening tests are most important for the early detection and successful treatment of a disease, especially if its progression leads to significant changes in the nature of underlying pathological processes. Cancers and neurodegenerative diseases are most common examples of such diseases. Cancer progression leads to tumor invasion into surrounding tissues, metastasis and clonal evolution, which dramatically complicates treatment (Richards, 2009; Caldas, 2012). Similarly, progression of neurodegenerative diseases, leads to a switch from metabolic abnormalities to neurite and synapse destruction and finally to irreversible neuronal death (Bredesen, 2009; Snyder et al., 2012). Further, disease progression leads to involvement of new brain areas and cell types in the pathology (von Bernhardi and Inestrosa, 2008). The new diagnostic methods based on imaging techniques and analysis of proteins and other components in the cerebrospinal fluid (CSF) have been developed recently (Apostolova et al., 2010; Fagan et al., 2011; Mori et al., 2012); these methods, however, are not suitable for the first line screening due to their invasiveness and relatively high cost.

## EARLY DIAGNOSIS OF NEUROLOGIC DISEASES

There are hundreds of brain disorders and many ways to classify them; however, from the viewpoint of diagnostics it is helpful to consider the diseases based on the need for different types of diagnostics, and on the underlying pathologic processes that need to be accurately and specifically detected by successful diagnostic tests.

First of all, all neurologic diseases fall into two large groups: acute and chronic pathologies. Obviously, there is a need in diagnostic and monitoring, but not screening tests for acute disorders, such as stroke, brain traumatic injury, and infections (of course, screening tests for predisposition to acute diseases, such as stroke, would be useful). On the other hand, numerous studies have demonstrated that, due to high compensatory potential of the brain, symptoms of chronic neurodegenerative diseases, such as Alzheimer’s (AD), Parkinson (PD), Huntington (HD) diseases, vascular and frontotemporal (FTD) dementias occur 10–20 years after the beginning of the pathology. The massive neuronal death, characteristic of late stages of neurodegenerative diseases, makes any therapeutic treatment late in the course of a disease extremely difficult, as illustrated by recent failures of anti-AD therapies in late stage clinical trials (Sperling et al., 2011; Pillai and Cummings, 2013). This phenomenon stresses the importance of the development of screening tests capable of detecting such diseases in early asymptomatic stage.

Further, it is instructive to group neurologic diseases according to the nature of the underlying physiological and pathological processes. For example, although molecular mechanisms of various neurodegenerative diseases are different, many processes, e.g., neurite retraction, dysfunction, and destruction of synapses, followed by neuronal death, are characteristic of neurodegeneration in general (von Bernhardi and Inestrosa, 2008); massive death of neurons and glial cells accompany initial stages of stroke and brain traumatic damage (McIntosh et al., 1998; Zheng et al., 2003); dysfunction of dopaminergic neurons is characteristic of addiction to many drugs (Kreek et al., 2012), etc.

Finally, physical location of pathological processes occurring during different stages of disease development is important for the focused search for specific biomarkers. The neurodegenerative diseases provide a good example of the diversity of physical locations involved: although many underlying processes are common for AD, PD, and FTD, initial pathological events characteristic for different diseases are localized in different brain areas: hippocampus for AD, midbrain for PD, and frontal lobe for FTD. At later stages, each disease spreads to new areas of the brain, which significantly worsens clinical symptoms and narrows treatment options; this expansion can in principle be used for disease monitoring.

A large number of studies focus on the development of minimally invasive molecular tests for early detection of AD, PD, and other brain pathologies. These include reports of some encouraging early data on the development of blood-based assays for AD diagnosis based on analysis of a large number of proteins or antibodies in human blood (Ray et al., 2007; Nagele et al., 2011; Reddy et al., 2011). In this review we describe new approaches to developing screening, diagnostic and monitoring tests for neurodegenerative diseases, and other brain pathologies based on analysis of cell-free microRNA in bodily fluids.

## ROLES AND PROPERTIES OF miRNA

miRNA is a class of non-coding RNA, whose final product is an approximately 22 nucleotide-long functional RNA molecule. miRNA repress translation and regulate degradation of their target mRNA by binding to complementary regions of messenger transcripts (Griffiths-Jones et al., 2006; Bartel, 2009). There are several programs for *in silico* analysis of complementarity between miRNA and mRNA; the lists of possible targets for a miRNA frequently include hundreds of genes. Hence, based on sequence analysis alone, a given miRNA can potentially be involved in numerous different pathologies (for example, see miR-Ontology Database: http://ferrolab.dmi.unict.it/miro/). All these predictions must be validated *in vivo*; in most cases this has not been accomplished yet, even though experimental data on miRNA roles in epigenetic regulation of numerous cellular processes are rapidly accumulating (Siegel et al., 2011; McNeill and Van Vactor, 2012). The following miRNA properties make them attractive for using in development of various diagnostic tests.

miRNA are small molecules with higher, e.g., compared to proteins, chances to cross blood–brain, placental, and other barriers and to appear in bodily fluids. Cell-free miRNA have been detected in plasma, serum, urine, saliva, and milk, where they are protected by membranes in exosomes and other microparticles, by various proteins, lipids and, possibly, other molecules (reviews: Sun et al., 2012; Zandberga et al., 2013). Although the organ, tissue, or cell origin of circulating miRNA is difficult to determine *in vivo*, it has been shown that many of those circulating miRNA do not originate in blood cells or cells present in or contacting with respective bodily fluids (Weber et al., 2010; Duttagupta et al., 2011). Numerous data on circulating miRNA have recently been assembled (Russo et al., 2012) and made available via miRandola database: .

More than 1,500 human miRNA have been discovered to date, and although there are no miRNA exclusively present in a single tissue or organ, many of them are highly enriched in particular organs, tissues, and cell types (Barad et al., 2004; Liu et al., 2004; Baskerville and Bartel, 2005; Beuvink et al., 2007; Landgraf et al., 2007; Liang et al., 2007; Wang et al., 2007; Castellano and Stebbing, 2013). Many of miRNA are enriched in the brain and, importantly for the development of diagnostic tests, various miRNA are enriched in different brain areas as well as in different cell types (neurons and glial cells; Sempere et al., 2004; Smirnova et al., 2005; Deo et al., 2006; Bak et al., 2008; Trivedi and Ramakrishna, 2009; Weng et al., 2011; He et al., 2012). Moreover, certain miRNA are present or even enriched in particular intracellular compartments, such as synapses, dendrites, and axons (Schratt et al., 2006; Lugli et al., 2008; Schratt, 2009; Edbauer et al., 2010; Natera-Naranjo et al., 2010; Strickland et al., 2011; Wu et al., 2011; Pichardo-Casas et al., 2012). A number of the listed above detailed studies of miRNA expression in various organs, tissue, and cell types have been performed prior to identification of many miRNA, and thus there is an obvious need for additional studies of expression profiles of these newly discovered miRNA. In particular, *in situ* hybridization studies would be very useful for mapping miRNA expression in different brain areas, cell types and intracellular compartments.

Intracellular concentration of miRNA changes in various physiological and pathological processes due to modifications in their transcription, maturation, and stability (Iorio and Croce, 2012). Such changes of miRNA levels in different brain areas are characteristic of many neurodegenerative diseases and other brain pathologies (reviews: Saugstad, 2010; Fiore et al., 2011).

A number of recent papers review the available data on changes in miRNA expression in different brain areas involved in AD development (Fiore et al., 2011). A comprehensive review by Tan et al. (2013) presents an analysis of the published evidence for the involvement of miRNA in the four processes playing critical roles in AD pathogenesis: accumulation of amyloid-β, tau toxicity, inflammation, and neuronal death. While some of the results appear compelling, many more studies are necessary to further elucidate the precise roles of individual miRNA in AD pathogenesis and their involvement in different stages of the disease. Some of the published data appear contradictory, possibly due to differences in methods employed for miRNA measurement and normalization in different studies. For example, both activation and inhibition of the brain-enriched miR-9 expression in hippocampus of AD patients have been reported (review: Jin et al., 2013).

Investigations of the miRNA involvement in PD have focused on analysis of miRNA expression in the midbrain and of miRNA role in functioning of dopaminergic neurons and the α-synuclein synthesis. Downregulation of miR-133b in midbrain of PD patients (Kim et al., 2007) as well as in mouse models of PD has been reported in several studies (reviews: Harraz et al., 2011; Filatova et al., 2012; Mouradian, 2012). miR-7 and miR-153 have been found to downregulate synthesis of α-synuclein (Junn et al., 2009; Doxakis, 2010); suppression of the expression of these miRNA in midbrain of PD patients is expected but has not been demonstrated yet.

Deregulation of 15 miRNA in the brain of the mouse model of prion-induced neurodegeneration has been demonstrated (Saba et al., 2008). Further, changes in miRNA expression caused by or at least accompanying schizophrenia, autism, cognitive dysfunction, drug addiction, neuroblastoma, and other neurologic disorders have been reported (review: Jin et al., 2013).

For the use of miRNA in diagnostics, it is also important that miRNA secretion varies depending on cellular physiology (Wang et al., 2009; Pigati et al., 2010; Palma et al., 2012). In addition to miRNA release into extracellular space and subsequent appearance in the bodily fluids due to cell death, miRNA appear in circulation due to blebbing of apoptotic bodies, budding and shedding of microvesicles, active secretion in the form of exosomes and of miRNA complexes with proteins (AGO2, NPM1, and others) and high density lipoproteins (HDL; reviews: Sun et al., 2012; Zandberga et al., 2013). All these forms of cell-free miRNA are highly stable in the bloodstream and other bodily fluids. The secretion of miRNA is selective and can be significantly changed by various pathological processes. For example, changes in the spectrum of miRNA secreted in exosomes from prion-infected neuronal cells, as compared to uninfected cells, have been demonstrated (Bellingham et al., 2012).

## IDENTIFICATION OF POTENTIAL miRNA BIOMARKERS IN BODILY FLUIDS

The development of screening and diagnostic tests for various diseases based on analysis of miRNA in bodily fluids – mainly in plasma or serum, but also in urine, saliva, CSF, and milk – is a very active area of research. Below are three approaches commonly used in such studies, including studies in the area of neurodegenerative diseases:

(1) Measurement of hundreds miRNA in a bodily fluid from patients with a pathology of interest and from control subjects using miRNA array or next generation sequencing (NGS; Qin et al., 2013). An obvious advantage of this approach is that huge numbers of various miRNA can be analyzed. Detection of miRNA sequence variations, which can be informative, e.g., for tumor diagnostics, is another advantage of NGS (Williams et al., 2013). Many potential biomarkers have been found by these techniques. However, the miRNA array-based and sequencing techniques are not sufficiently sensitive to detect many miRNA whose concentration in bodily fluids is relatively low. Usually only 30–40% of plasma or serum miRNA detectable by an individual RT-PCR is measurable by various miRNA arrays. This is also true for the RT-PCR-based array, largely due to significantly lower amounts of plasma analyzed by array compared to individual RT-PCR. In addition, reproducibility is very important for screening tests addressing early disease stages when changes in biomarker concentrations usually are not high. Unfortunately, variability of miRNA array tests is significantly higher than that of the individual miRNA RT-PCR (Leidner et al., 2013). As a consequence, potential biomarkers selected by array analysis have to be confirmed by RT-PCR, and indeed in many cases the array data is not confirmed. Further, even when miRNA array-selected potential biomarkers are validated by RT-PCR, they cannot be automatically considered useful for screening or diagnostic purposes. Most of the miRNA detectable in bodily fluids by arrays are ubiquitous miRNA expressed in all or many tissues, and many of them derive from blood cells (Pritchard et al., 2012; Leidner et al., 2013); the detection of changes in their concentrations in patients with one pathology does not mean that the same miRNA cannot be involved in other diseases of different organs. Many miRNA are associated with a particular pathology type, such as cancer, inflammation, hypoxia, etc., and changes in their concentration in bodily fluids can be associated with diseases of different organs. For example, changes of miR-155 concentrations were found in the bloodstream of patients with breast, esophageal, lung, pancreatic cancers and lymphomas (Blair and Yan, 2012; Xie et al., 2013). Level of miR-21 increases in plasma/serum of patients with osteosarcoma, bladder, esophageal, gastric, lung, breast, colorectal cancers, neck squamous cell carcinoma, and other tumors (Farazi et al., 2011; Blair and Yan, 2012; Xie et al., 2013). It follows that the potential biomarkers found by miRNA arrays should be also tested in other pathologies, not only in healthy control subjects.
(2) The second approach is based on analysis of disease-specific miRNA identified by comparison of miRNA isolated from pathologic and normal tissue, organ, or cell type. Here, subsequent to identification of disease-specific miRNA, e.g., by array followed by RT-PCR, their presence in bodily fluids is analyzed. There are obvious advantages to this strategy. First, a limited number of circulating miRNA should be tested, which makes the use of individual RT-PCR appropriate, thus increasing sensitivity and reproducibility of the analysis. Second, an observation of a correlation between changes in miRNA concentrations in a bodily fluid and in an organ involved in pathology directly suggests that the miRNA is a suitable biomarker for screening tests. However, such a correlation does not always exist and sometimes miRNA concentrations in a bodily fluid and in a target organ change in opposite directions (Boeri et al., 2011; Cuk et al., 2013). This phenomenon can be explained by several factors: (i) if pathology is caused by, or associated with, the change in concentration of a ubiquitous miRNA, the effect of the pathology on the concentration of this miRNA in circulation could be very limited, since only a small fraction of the miRNA in circulation comes from the affected organ or tissue; (ii) changes in miRNA concentration due to pathology development can be accompanied by much more prominent opposite changes in miRNA secretion/excretion, which neutralizes or even overcomes the effect of changed miRNA expression. Of course, these limitations do not preclude successful use of the described approach for the discovery of circulating miRNA biomarkers. In the area of neurodegenerative and other neurologic pathologies this approach can be especially productive for the miRNA biomarker discovery in CSF (see below) because: (i) CSF is not separated from brain by the blood–brain barrier; and (ii) the amount of miRNA secreted or excreted from other organs to CSF is very limited (Cogswell et al., 2008).
(3) The third approach was proposed recently for searching for biomarkers of mild cognitive impairment (MCI), AD, and other neurodegenerative diseases (Sheinerman et al., 2012; Sheinerman and Umansky, 2013). It is based on analysis of circulating brain-enriched miRNA in bodily fluids, e.g., in plasma. Restricting the analysis to brain-enriched miRNA significantly increases the chances that any observed changes in concentrations of these miRNA in plasma are caused by brain-related processes. In addition, it is proposed to include miRNA enriched or at least present in neurites and synapses, i.e., neuronal compartments, which are involved in neurodegenerative diseases from early stages of pathology development, years before massive death of neurons. It could be also useful to search for miRNA enriched in the brain area, which suffers first during development of a particular disease, e.g., in hippocampus for AD and in midbrain for PD potential biomarkers. As a number of miRNA to be investigated is limited, RT-PCR can be used here, which is critically important since plasma concentration of many brain-enriched neurite/synapse miRNA is too low to be reliably detectable by miRNA arrays. The advantages and disadvantages of the three approaches described above are summarized in Table 1.

**Table 1:** Current approaches to analysis of miRNA in plasma. The same approaches can be used for miRNA analysis in different body fluids (urine, saliva, and milk).

miRNA array/NGS	Disease-specific analysis	Organ/cell-specific analysis
**Steps**
1. miRNA isolated from plasma 2. Array analysis 3. RT-PCR verification 4. Validation	1. miRNA isolated from pathologic and normal tissue 2. Array analysis 3. RT-PCR verification 4. Selected miRNA analyzed in plasma 5. Validation	1. miRNA isolated from plasma 2. RT-PCR analysis of organ, cell-enriched miRNA 3. Validation
**Advantages**
• 100s of miRNA tested	• Disease-related miRNA tested • High sensitivity	• Organ/cell-specific miRNA tested • High sensitivity
**Disadvantages**
• Low sensitivity • High variability	• Same miRNA can be involved in pathologies of various organs • miRNA levels in tissue and plasma do not always correlate • Selected miRNA can be undetectable in plasma	• miRNA biomarkers not enriched in target • organs or cells can be missed

## NORMALIZATION OF miRNA CONCENTRATION IN BODILY FLUIDS

The concentration of miRNA detected in bodily fluids depends on many biological and technical factors. Biological factors include miRNA levels in various tissues, intensity of secretion and excretion into extracellular space, forms of circulating miRNA (exosomes and other vesicles, complexes with proteins and lipids) affecting their ability to cross various barriers, e.g., blood–brain, placental, and kidney barriers, and miRNA stability and half-life in the bloodstream. Technical factors include variability during bodily fluid collection and storage, methods used for miRNA extraction, and the presence in bodily fluids of various factors affecting miRNA purification and RT-PCR. As a consequence, the importance of miRNA normalization is currently broadly recognized (Meyer et al., 2010). At the same time no single normalization method is commonly accepted. Several methods of circulating miRNA normalization have been described in literature:

(1) Normalization per spiked miRNA, which is absent in the investigated species, e.g., *C. elegans* or plant miRNA absent in mammalian cells (Sarkar et al., 2009; Kroh et al., 2010; Sanders et al., 2012). Such miRNA is spiked into a bodily fluid after addition of a lysing solution that inhibits nuclease activity and then is used as a normalizer by the ΔCt approach compensating for variability caused by RNA extraction and the possible presence of RT-PCR inhibitors.
(2) Normalization per ubiquitous and least variable circulating miRNA (Peltier and Latham, 2008; Latham, 2010; Lardizábal et al., 2012). This method is used for comparing circulating miRNA concentrations in controls and in one or a relatively small number of pathologies, which are not associated with changes of a normalizer miRNA. For example, miR-16 is widely used as such normalizer (Kroh et al., 2010). One needs to be mindful, however, that miR-16 is involved in regulation of apoptosis and its expression is changed in many pathologic processes, which leads to changes of its concentration in plasma and other bodily fluids (Schaefer et al., 2010; Katsuura et al., 2012; Wang et al., 2012b). Similar considerations apply to other miRNA normalizers of this type.
(3) Normalization per other (not miRNA) small RNA, mainly small nuclear or small nucleolar RNA. This approach was often used in earlier studies of circulating miRNA, and is still used although more rarely (Sanders et al., 2012). In addition to problems similar to the ones described in the previous paragraph (changes in the RNA concentration associated with some pathologies),the utility of this approach is limited by the fact that these RNA are larger than miRNA, bound to different proteins, and their stability is different from that of miRNA (Lardizábal et al., 2012).
(4) If a large number of miRNA is analyzed, usually by miRNA arrays, normalization per average of all miRNA can be used (Mestdagh et al., 2009; D’haene et al., 2012; Blondal et al., 2013).
(5) Finally, in recent years application of “miRNA pair” approach is becoming increasingly popular (Boeri et al., 2011; Hennessey et al., 2012; Matthaei et al., 2012; Sheinerman et al., 2012). In this case, ratios (ΔCt) of each miRNA to each other miRNA measured in a given experiment is calculated, and pairs, which provide the highest accuracy in differentiating patients with a disease of interest from controls, are selected. A variant of the approach is based on the use of miRNA enriched in the organ of interest (Sheinerman et al., 2012; Sheinerman and Umansky, 2013). The rationale for the approach is as follows. First, any pathology is usually associated with upregulation of some miRNA and downregulation of other miRNA, thus considering miRNA pairs may increase test sensitivity and specificity. Second, use of the pair of, rather than one, miRNA enriched in the same organ decreases potential overlap with pathologies of other organs. Third, one can expect that changes unrelated to or non-specific for a pathology of interest, such as changes in blood supply, blood–brain permeability (for neurological pathologies) and others, will be better compensated for by using the pair of miRNA enriched in the same organ. This approach is particularly promising for neurodegenerative and other neurologic diseases. For example, combination of neurite/synapse miRNA with a neuronal body miRNA may be informative for evaluating disease progression from synapse destruction to neuronal death. The changes in relative concentrations of miRNA enriched in different brain areas or different cell types (e.g., neurons and glial cells) may be an indicator of disease progression, and so on.

In summary, data normalization is an extremely important step in developing tests based on analysis of circulating miRNA, and as such it represents an active area of research.

## SEARCH FOR miRNA BIOMARKERS FOR NEUROLOGIC DISEASES

There are numerous recent reviews on miRNA role in brain development, normal brain aging, and various neurological disorders (Krichevsky et al., 2003; Gascon and Gao, 2012; Mellios and Sur, 2012; Salta and De Strooper, 2012; Smith-Vikos and Slack, 2012; Wang et al., 2012a; Xu et al., 2012; Dimmeler and Nicotera, 2013; Moreau et al., 2013). Here we concentrate on circulating cell-free miRNA as potential biomarkers for diagnosing neurologic diseases. Despite well-recognized need in early detection of neurodegenerative diseases and other neurological disorders and great promise of circulating miRNA as potential biomarkers, there are relatively few publications in the area. There could be several explanations for this phenomenon, including an observation that concentration of many circulating cell-free brain-enriched miRNA in plasma, serum, and other bodily fluids is relatively low and thus they are not easily detectable by commonly used miRNA arrays. As mechanisms of miRNA appearance in CSF and bloodstream are different, below we separately describe the existing data for these two bodily fluids.

### CELL-FREE miRNA IN CSF AND CNS DISORDERS

The presence of miRNA in CSF was first demonstrated by Cogswell et al. (2008). Cogswell et al. (2008) also reported changes in levels of many miRNA in CSF of AD patients when compared to controls. The authors came to a conclusion, however, that the major source of miRNA detected in CSF are immune cells present in CSF since “there was no obvious relationship between the miRNAs altered in CSF and the absolute levels in sites of AD mediated destruction or the directional changes in those regions.” Many miRNA, including those that are highly enriched in brain, were not detectable in CSF by methods used. In another study devoted to the same matter the increase in levels of pro-inflammatory miR-146a and miR-155 in CSF of AD patients compared to age-matched controls as well as upregulation of neuron-enriched miR-9 and miR-125b was reported (Alexandrov et al., 2012). Interestingly, Lukiw et al. (2012) also demonstrated upregulation of miR-145b and miR-155 in human neuronal–glial primary cocultures 12 and 36 h after stress treatment and proposed that extracellular miRNA can be involved in spreading of AD inflammatory signaling.

Several studies describe changes in concentration of CSF miRNA in patients with different brain tumors. miR-15b and miR-21 were differentially expressed in CSF from patients with gliomas, compared to controls with various neurologic pathologies, including patients with primary CNS lymphoma and carcinomatous brain metastasis (Baraniskin et al., 2012). The changes in expression and plasma/serum levels of both miR-15b and miR-21 are commonly associated with different types of cancers and are not specific for gliomas. However, it seems unlikely that their concentrations in CSF can be strongly affected by non-CNS tumors. This conjecture needs to be proven for these miRNA to be used as glioma biomarkers. Teplyuk et al. (2012) found a significant increase of miR-10b and miR-21 levels in CSF of patients with glioblastoma and brain metastasis of breast and lung cancers, compared with various non-neoplastic conditions, such as memory problem, dementia, PD, encephalitis, and others. These two miRNA were also significantly upregulated in glioblastoma, compared to normal brain. The use of CSF levels of seven miRNA – miR-10b, miR-21, miR-125b, miR-141, miR-200a, miR-200b, and miR-200c – permitted the authors to achieve high accuracy in separation between all classes of samples. Again, only miR-125b from this list is a brain-enriched miRNA; all other miRNA are associated with carcinogenesis itself, so their usefulness as potential biomarkers is based on their presence in CSF, which is isolated from tumors located in other organs outside of CNS.

Microparticles containing miRNA, including brain-enriched ones, have been found in CSF and the spectrum of miRNA detectable in CSF is changed after brain injury (Patz et al., 2013). miR-451 was detected in CSF microparticles only after brain injury, which can be explained by more effective secretion of this miRNA from abnormal cells (Pigati et al., 2010).

### CELL-FREE miRNA IN PLASMA AND SERUM AND CNS DISORDERS

Due to massive damage and death of neurons and glial cells the concentration of circulating brain-enriched miRNA is dramatically increased in the bloodstream after a stroke. For example, in the rat model, the plasma level of miR-124 is up to 150 times higher than in plasma of sham-operated animals (Laterza et al., 2009). Of course, one cannot expect such large changes of miRNA biomarker levels in plasma/serum from patients with chronic, slowly developing neurodegenerative diseases, such as AD. Perhaps, this consideration explains why, in spite of the huge need in a minimally invasive test for early detection of AD, there are only two publications devoted to the use of circulating miRNA for diagnosis of this pathology.

Geekiyanage et al. (2012) compared concentrations of five miRNA, namely miR-137, miR-181c, miR-9, miR-29a, and miR-29b in serum of MCI and AD patients with their levels in serum of age-matched controls. The choice of miRNA was based on the previous study (Geekiyanage and Chan, 2011) that demonstrated their involvement in AD pathogenesis and downregulation of these miRNA in the brain cortex of sporadic AD patients. Using RT-PCR and normalization per spiked cel-miR-39 and internal miR-22, miR-191, and miR-126 the authors demonstrated that the levels of miR-137, miR-181c, miR-9, miR-29a, and miR-29b are significantly lower in serum of MCI and AD patients, compared to controls. Since each group of patients and controls included only seven subjects, sensitivity and specificity of MCI and AD detection could not be calculated.

Sheinerman et al. (2012) investigated the use of circulating miRNA for early detection of MCI. Since neuronal death, a late event in the development of AD and other neurodegenerative diseases, is preceded by metabolic changes, neurite retraction, synaptic dysfunction, and synapse loss, the authors suggested that these processes could cause excessive secretion and excretion of miRNA from brain areas involved in the pathology. Thus, brain-enriched miRNA, including neurite- and synapse-enriched miRNA were measured in plasma of MCI and AD patients and age-matched control subjects. Described above “miRNA pair” approach was used for data normalization. In the preliminary experiments 32 miRNA were analyzed, then 13 most promising miRNA were selected for the feasibility study. Finally, two sets of biomarker miRNA pairs were identified: the “miR-132 family” (miR-128/miR-491-5p, miR-132/miR-491-5p, and mir-874/miR-491-5p) and the “miR-134 family” (miR-134/miR-370, miR-323-3p/miR-370, and miR-382/miR-370). Each biomarker pair included as numerator neurite/synapse-enriched miRNA. These potential biomarkers differentiated MCI from age-matched controls with sensitivity and specificity of 79–100% (miR-132 family) and 79–95% (miR-134 family). In a small longitudinal study (19 subjects), the identified miRNA biomarker pairs successfully detected MCI in majority of patients at asymptomatic stage 1–5 years prior to clinical diagnosis. MCI is a heterogeneous syndrome. On average, 10–15% of MCI patients annually convert to dementia. About 80% of dementias are caused by AD and the rest of dementia patients are diagnosed with other neurodegenerative diseases. It is perhaps not surprising then that both sets of miRNA pairs also differentiated AD from age-matched control. The biomarkers do not distinguish AD from MCI, however, indicating that these biomarker miRNA pairs detect processes characteristic of various neurodegenerative pathologies (synapse destruction?) but not AD-specific events. Interestingly, these biomarker pairs also appear useful for detecting age-related brain changes. The reported results await confirmation in larger clinical studies.

One study (Gaughwin et al., 2011) described a search for a plasma miRNA, which could be used for early diagnosis of HD. The authors tested many miRNA regulated by mutant HTT protein and found that one of these miRNA, miR-34b, is significantly elevated in plasma from carriers of mutant HTT prior to symptom onset. However, it is unclear if miR-34b can be a specific biomarker for HD, since this miRNA is expressed in many tissues, being especially enriched in pulmonary system, Fallopian tubes, and testicles, and is also deregulated in various pathologies.

Several groups (Haghikia et al., 2012; Siegel et al., 2012; Gandhi et al., 2013; Ridolfi et al., 2013) reported promising results regarding potential use of circulating miRNA in CSF or bloodstream for diagnosis of multiple sclerosis (MS). Since MS is an autoimmune neurodegenerative disease, one can expect that concentration of both brain-enriched miRNA and of inflammation and immune system-associated miRNA could be changed in CSF and plasma/serum. Currently, the majority of the discovered potential biomarkers belong to the latter group: inflammation and immune system associated miRNA.

A limited number of studies report analyses of circulating miRNA in plasma or serum of patients with various neurological and psychological disorders, such as depression (Li et al., 2013), bipolar disorder (Rong et al., 2011), and schizophrenia (Shi et al., 2012), suggesting that the study of circulating miRNA in plasma/serum for diagnosis of neurological disorders represents a promising direction for further more detailed investigations.

## SUMMARY AND PERSPECTIVES

In spite of the rapidly growing number of publications on diagnostic applications of circulating cell-free miRNA, their use for screening of CNS diseases is in early stages of development.

One factor impeding the progress in the field is the difficulty of comparing the data reported by different groups due to the use of different methods for searching for potential circulating miRNA biomarkers, different techniques for miRNA measurement and data normalization. Broad acceptance of best practices and uniform methods for analysis of circulating miRNA and the development of statistical apparatus for comparing the results obtained by different techniques will be important to overcome the problem.

Further, when miRNA arrays are used for initial miRNA screening, many tissue-enriched miRNA are not detectable and, as a consequence, ubiquitous miRNA, including miRNA associated with common pathologic processes such as carcinogenesis or inflammation, are often selected as potential biomarkers. These miRNA can successfully differentiate patients with a particular disease from healthy control subjects, but not necessarily from patients with similar pathologies of other organs, since it is highly likely that the plasma or serum levels of these miRNA will also be affected in such patients. From this perspective, searching for miRNA biomarkers in CSF has advantages for detecting CNS disorders, similar to advantages of using stool miRNA for detecting colon diseases (Ahmed et al., 2013): in each of these cases miRNA come from the organ of interest. The invasiveness of CSF collection, however, precludes using it for primary screening, and thus emphasis needs to be placed on the development of screening tests based on analysis of plasma or serum.

An analysis of data in literature suggests a number of optimization steps to increase the chances of success for such efforts. First, the use of brain-enriched miRNA for CNS disorders should increase biomarker specificity. This thesis is supported by data obtained for other organs. For example, increase in plasma/serum concentration of liver-enriched miR-122 is very common in patients with pathologies of this organ (Zhang et al., 2010), and increase in plasma/serum concentration of heart-enriched miR-1, miR-133a, miR-133b, or miR-499-5p is characteristic of acute myocardial infarction and some other cardiac pathologies (Tijsen et al., 2012). Second, combination of several circulating miRNA biomarkers can increase test specificity, since even expression of ubiquitous miRNA varies from organ to organ and one can expect that spectrums of concentration of several miRNA biomarkers will be different for various pathologies of different organs. For example, Li et al. (2011) made an interesting observation of the age-dependent increase of miR-34a concentration in brain, peripheral blood mononuclear cells, and plasma. This miRNA, however, cannot be used as a sole biomarker of aging, since its concentration in plasma is changed in patients with tumors of various locations and with other pathologies. At the same time, in combination with mir-132 and miR-134 biomarker families described above, this miRNA could be a helpful additional biomarker. Finally, it can be useful to combine tissue-enriched miRNA with miRNA associated with a common pathology type, e.g., carcinogenesis.

The data reviewed herein suggests strongly that the analysis of cell-free miRNA in bodily fluids is a highly promising approach for developing minimally invasive screening tests for CNS disorders. A different question is whether circulating miRNA represent a promising class of molecules for the prognosis of neurological disease outcomes and for disease and treatment monitoring. Naturally, diagnostic biomarkers can be used for disease monitoring if same pathologic processes underlie the development of the disease during different disease stages. The use of diagnostic biomarkers for disease monitoring is more problematic, however, if disease progression is caused by involvement of new pathologic processes. AD is a good example of such a disease: (i) the pathology is initiated by not fully understood metabolic abnormalities, (ii) these are followed by morphological changes in particular brain areas, such as formation of amyloid plaques and tau protein tangles, neurite retraction, dysfunction, and destruction of synapses, (iii) finally neurons are dying, and pathology expands to new brain areas and cell types. Thus, it is unlikely that different stages of AD can be differentiated by the same biomarkers. The situation is also complicated by the fact that some processes, e.g., synapse destruction, are common for different pathologies, including normal brain aging and other neurodegenerative diseases. The detection of such common processes is clearly useful for monitoring of normal brain aging and diagnosis of MCI, which is a syndrome characteristic of early stage of various neurodegenerative diseases. However, such a test will not predict MCI outcome. This goal could be accomplished by other tests, such as CSF protein analysis and/or imaging techniques, or by different miRNA biomarkers specific for various AD stages. For example, AD expansion to new brain areas and cell types can be monitored by circulating miRNA enriched in those brain areas and cell types. The switch from synapse destruction to neuronal death can potentially be detected by changes of ratios in plasma of cell-free synapse-enriched and neuronal body miRNA, and so on. Of course, all such proposals need be tested, preferably in longitudinal studies.

We hope the advances in the analyses of circulating miRNA reviewed here will lead to more efforts toward using miRNA as biomarkers of neurodegeneration, of other neurologic pathologies, and of normal brain aging, and will facilitate the development of screening, predictive, and monitoring tests for these diseases.

## Conflict of Interest Statement

Kira S. Sheinermanand Samuil R.Umansky are shareholders of DiamiR, LLC.
